# A Two-step Approach for Interest Estimation from Gaze Behavior in Digital Catalog Browsing

**DOI:** 10.16910/jemr.13.1.4

**Published:** 2020-04-01

**Authors:** Kei Shimonishi, Hiroaki Kawashima

**Affiliations:** Kyoto University, Japan; University of Hyogo, Japan

**Keywords:** decision making, eye movement, aspect-model, region of interest, gaze, attention, interest

## Abstract

While eye gaze data contain promising clues for inferring the interests of viewers of digital catalog content, viewers often dynamically switch their focus of attention. As a result, a direct application of conventional behavior analysis techniques, such as topic models, tends to be affected by items or attributes of little or no interest to the viewer. To overcome this limitation, we need to identify “when” the user compares items and to detect “which attribute types/values” reflect the user’s interest. This paper proposes a novel two-step approach to addressing these needs. Specifically, we introduce a likelihood-based short-term analysis method as the first step of the approach to simultaneously determine comparison phases of browsing and detect the attributes on which the viewer focuses, even when the attributes cannot be directly obtained from gaze points. Using probabilistic latent semantic analysis, we show that this short-term analysis step greatly improves the results of the subsequent step. The effectiveness of the framework is demonstrated in terms of the capability to extract combinations of attributes relevant to the viewer’s interest, which we call aspects, and also to estimate the interest described by these aspects.

## Introduction

Estimating the real-time interest of users browsing a digital catalog opens a variety of application possibilities including online recommendation of items that might better fit their needs and automated assistance, such as offering a new viewpoint for their choice [1, 2, 3]. Bring such systems into reality requires the development of a representation of user interest and a method for estimating it. 

Consider a situation in which a user is browsing a digital catalog containing items, each with multiple attributes, and selects one item. According to the concept of means-end chains [4, 5], it is desirable to estimate user interest not only on individual items and attributes but also on the user’s personal values because such values are often linked to the basic reason for a choice. 

**Figure 1.**
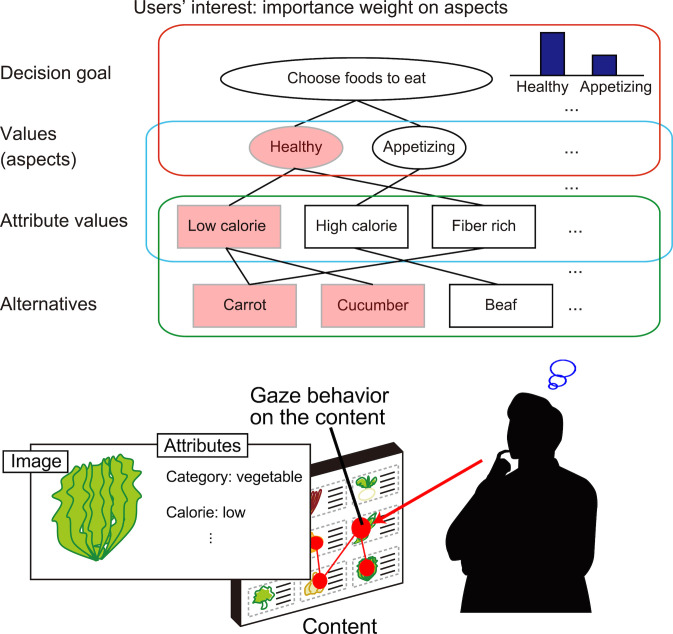
Example situation of users’ choice behavior during content browsing and user’s internal hierarchical structure based on means-end chain concept.

To represent user values, we assume that “each value can be associated with a subset of attributes.” For example, the value “health” has strong relevance to “low calorie” and “fiber rich” attributes. A model of a user’s internal process for this situation can be illustrated in Figure 1, which is based on the means-end chain concept. By assuming that personal values can be defined as certain aspects of items in a content domain, we introduce *aspects *as the representation of personal values. 

In the course of automated inference of user interests in this setting, a set of aspects needs to be prepared beforehand to represent personal values. In fact, the successful estimation of user interest depends on the appropriateness of the prepared aspects; meanwhile, they depend on many factors, such as content domains, the user’s characteristics, and the task being engaged [6]. As will be discussed in the related work section, one approach to the analysis is to use interviews, but the quality of the data collected depends on the interviewer’s skills; moreover, there is a cost involved. 

In this paper, we therefore investigate another, data- driven, approach to obtaining a set of aspects from users’ behavior by assuming that “users share several common aspects related to the same content domain.” While such an assumption may not always be valid, it is still useful, at least for actual applications of decision support. 

### Research objective

This paper addresses two problems related to using eye gaze data collected during digital catalog browsing: (1) data-driven extraction of aspects that describe user interest and (2) estimation of the user interest. 

The analysis of sensory information (e.g., GPS coordinates, click streams) with machine-learning techniques, such as topic models, is a growing trend for identifying an association between a user’s behavior and the user’s internal state [7]. Among these techniques, eye tracking is a promising approach to closely exploring a user’s internal states during decision making [8, 9].

Our preliminary experiments revealed two important observations related to the limitation of directly applying topic models to gaze data: 

Observation 1. Users frequently switch their browsing states, e.g., from “simply grasping information about items” to “actively comparing items based on their interest”;

Observation 2. Users do not always take into account all the attributes of displayed items but rather focus on a subset of them. 

In a large-scale analysis of users’ click histories on websites, Das et al. found that user clicks are noisier than their explicit ratings and purchase activities [10]. That is, a user’s browsing behaviors (e.g., eye gazes and clicks) are not always closely associated with the user interest. In fact, eye gazes can be much noisier than clicks because gaze data capture a wide range of the human decision-making processes behind clicks. Additionally, each gaze point is only a slice of the user’s information processing in contrast to click activities, which involve more explicit decisions to obtain additional information. Therefore, it is likely that users focus on only some of the attributes of items on which gaze points are located. 

As a result, the aspects obtained from the direct application of topic models tend to be affected by items or attributes of no interest to the user. To overcome this limitation, we need to identify “when” the user compares items and to detect “which attribute types/values” reflect the user’s interest. 

This research aims at providing a novel approach to obtaining a set of aspects from user gaze data collected during content browsing by extracting the dynamic changes in the user’s “focus” on attributes. It also aims at determining how the automatically obtained aspects can be used to represent and estimate the user interest. 

### Contributions

**Figure 2.**
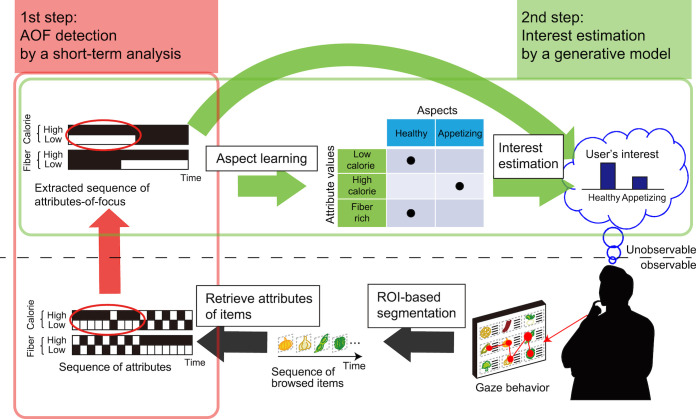
Flow of the two-step approach for interest estimation from user’s gaze behavior: 1st step is to detect user’s attributes-of-focus by applying likelihood-based short-term analysis; 2nd step is to estimate user interest by applying a probabilistic.

This paper proposes a novel two-step approach to the analysis of eye gaze data for aspect learning and interest estimation as described in the research objective above (the flow of the approach is summarized in Figure 2). 

In the proposed framework, a user’s gaze behavior is interpreted as the sequence of items at which the user looked (the bottom right part of Figure 2). The sequence of attribute values of each attribute type is retrieved from the sequence of items (the bottom left part of Figure 2). From the sequence of attribute values, as the first step, we introduce a likelihood-based short-term analysis of the attribute values at which the user looked (the red-highlighted area in Figure 2) for detecting distinctive gaze behavior while the user is actively comparing items. At the same time, the attribute values on which the user focuses during the distinctive periods are also extracted, which we refer to as the *attributes-of-focus *(or AOF for short). An example of AOF detection is shown in Figure 2 by the red-circled period when the user focuses only on “low calorie” and does not focus on any other attributes. As the next step, we apply a probabilistic generative model to the AOF (the green-highlighted area in Figure 2), a variant of the probabilistic latent semantic analysis (pLSA) model (topic model), to obtain the aspects and also to estimate the user interest described by the aspects.

The generative model used in the second step is an extension of our previous work [11], which took all attribute values of items into account. In contrast, the basic idea now is to use the AOF behind the gaze behavior as the “observation” of the subsequent probabilistic generative model. This is done by applying the first step. Along with introducing the first step, the generative model is modified to handle the AOF. While gaze behavior data is much noisier than the intended actions (e.g., explicit rating) used in traditional topic models, the first step acts as a filter to distinguish meaningful gaze data from the original data. 

### Organization of this paper

In the following two sections, we briefly review related work and introduce the details of the two-step approach: a likelihood-based short-term analysis for AOF detection and a generative model for aspect learning and interest estimation. We then evaluate our framework, discuss limitations, and conclude in the subsequent sections. 

## Related Work

### Analysis of values behind decision making

The means-end chain model [5] is one of the well-known methods for analyzing value-oriented behaviors in decision making [6, 12] and exploring consumer motivations [13, 14]. In this model, consumer decision making consists of options (means), consequences, and values (ends), and the model explains how a product can achieve the desired end states. Note that, in this paper, we use the term “value” to also describe “desired consequence” for simplicity, whereas the different levels are distinguished in means-end chain models. To apply the means-end theory, an interview-based method, such as laddering, is widely utilized in marketing research [13, 14, 15]. In laddering methods, interviewers directly ask users about the values driving their decision making. Since the user responses are diverse, the use of laddering to obtain appropriate values faces several challenges [16]. These challenges include the need to elicit information about the user’s values and the need to control the dialog; therefore, the results strongly depend on the interviewer’s skills [15]. In addition, many interviews are needed for each decision domain to obtain a sufficiently large value set. 

This difficulty is also seen in the analytic hierarchy process (AHP) [17], which is an organization technique for group decision making. The AHP structures a decision problem similarly to the means-end chain model. Participants in the decision-making process first discuss criteria for the problem and then construct a hierarchical structure consisting of a decision goal, goal alternatives, and the criteria. Once a hierarchical structure for decision making is constructed, the alternatives can be sorted with respect to the weights of each of the criteria. However, the quality of the construction step depends on the skill and knowledge of the participants. 

### Estimation of internal states behind user behavior

 Estimation of a user’s internal states from the user’s behavior is attracting researchers’ attention thanks to improvements in automated data acquisition technologies (e.g., sensors and the Web). For example, search logs on the Web contain rich information from which one can infer a user’s internal states [18, 19, 20, 21].

While the inference of internal states requires the representation of the states (i.e., state space), it is often difficult to manually prepare the state space itself since appropriate representation depends on the situation. Unsupervised machine learning techniques, such as topic models (latent factor models) [21, 22, 23, 24, 25], are widely studied as promising techniques for finding a representation of a user’s internal states from his or her decision-making behavior. Iwata et al. [22], for example, proposed a model for estimating temporal changes in consumer interest and item trends and tracking time-varying item trends by analyzing item purchase logs using a variant of the dynamic topic model. 

### Internal and external factors of gaze behavior

 Human visual attention is affected by both internal and external factors [26, 27, 28], which direct a user’s goal-oriented and stimulus-oriented attention, respectively. 

Goal-oriented attention is driven internally by one’s goals, so the resultant gaze behavior is affected by the task being engaged even if the same stimulus is presented [29, 30]. In fact, a number of studies have been conducted on cognitive-state estimation from eye gaze. The applications developed include inferring a user’s knowledge levels [31], a user’s cognitive ability to read graphs [32], and a user’s engagement in conversations [33]. A user’s gaze behavior has also been used to estimate his or her preference from content browsing [34, 35]. For example, Brandherm et al. developed an approach for estimating a user’s preferred target in displayed content on the basis of the frequency and duration of gazing at targets [34].

Stimulus-oriented attention is directed by external factors, such as the visual saliency of a scene [36]. Therefore, the effects of external factors also need to be considered when analyzing gaze behavior during choice. For example, the effect of spatial position is known to be large, especially after short-duration presentation of a visual target (e.g., an image) [37]. Furthermore, external factors themselves can even change the decision results. For example, Milosavljevic et al. reported that salient targets tend to be chosen when the decision time is short or when a cognitive load exists [38].

### Gaze behavior and decision phase

User decision making consists of several phases [39, 40] such as browsing a catalog to acquire information (screening phase) and comparing items to evaluate them (comparison phase). Note that the comparison phase is expected to contain more clues to a user’s values than the screening phase. To identify the decision phases, a short-term (segment-wise) analysis of gaze region sequences has been proposed [40, 41]. A tri-gram of gaze region sequences is considered to be a unit of analysis to extract layout-related gaze features for classifying the decision phases (browsing states). 

In this work, we focus on analyzing comparison behavior rather than rapid choice behavior by explicitly discriminating the screening and comparison phases. Using this experimental design, we analyze gaze behavior in the comparison phase since the effect of visual saliency in this phase is reduced due to the proceeding screening phase, as will be explained in the evaluation section. 

## Methods

### Two-step approach to estimating user interests

We first introduce the representation of content and user interest used to describe the decision-making situation for a digital catalog. Let $I=\{I_{1},\ldots ,I_{N}\}$ be a set of items in a digital catalog and 𝒜 be a set of attribute types common to all the items, where every item takes one attribute value from the set of attribute values $V^{\left(a\right)}=\{V_{1}^{\left(a\right)},\ldots ,V_{K_{a}}^{\left(a\right)}\}$ for each attribute type $V^{\left(a\right)}=\{V_{1}^{\left(a\right)},\ldots ,V_{K_{a}}^{\left(a\right)}\}$ . For example, “calorie” is an attribute type, while “high” and “low” are its values. 

To represent the user interest, we introduce *aspects* of items to describe possible reasons for comparison. Let $C=\{C_{1},\ldots ,C_{R}\}$ be a set of R aspects of items in a content domain, where these aspects are assumed to depend not on the user or displayed item set but on the content domain, as mentioned in the introduction section. We also assume that each aspect can be characterized by its association with attribute values. For example, the aspect “healthy” in the food content domain is relevant to “low calorie” and “fiber rich.” We model an aspect $C_{r}$ using parameter $p_{r}=\left(p_{r,1},\ldots ,p_{r,K}\right)(p_{r,k}\geq 0,\sum_{k}p_{r,k}=1)$ , where $K=\sum_{a\in A}K_{a}$ is the total number of attribute values, and each element denotes the degree of association with each attribute value. Corresponding to the representation of the aspect, we also denote all attribute values as $\left\{V_{1},\ldots ,V_{K}\right\}$ without the indicator of attribute type a for convenience.

Given the aspects introduced above, we model a user’s interest as an R -dimensional parameter vector $\theta =\left(\theta_{1},\ldots ,\theta_{R}\right)(\theta_{r}\geq 0,\sum_{r}\theta_{r}=1)$ , where each element of θ corresponds to the importance weight on each aspect. We assume that a user’s interest θ remains constant during each session, where “session” denotes one decision-making period involving browsing behavior for choosing one item. The goal of this paper is to estimate a user’s interest θ(s) for session s by learning ${\left\{p_{r}\right\}}_{r}=\{p_{1},\ldots ,p_{R}\}$ , the degree of association between aspects and attribute values, from the data collected on the user’s gaze behavior. 

The main idea of our approach is the explicit use of AOF, i.e., the attributes upon which the user actually focused. Although each item has attribute values for all attribute types a∈A , only a subset of attributes is taken care of in a decision-making session. We therefore introduce a designated step to extract AOF before applying a probabilistic generative model for interest estimation using unsupervised learning. 

Our approach thus consists of two steps (Figure 2). As the first step, we apply a likelihood-based short-term analysis to the sequences of gaze targets, i.e., the regions of interest (ROIs). In this step, the AOF are extracted by detecting the periods of distinctive gaze behavior, which are characterized by biased gaze-target patterns from neutral browsing. As will be shown later, this step is simple but highly effective for the subsequent second step, learning aspects and estimating user interest from gaze behavioral data using a generative model. As a concrete model for the second step, we propose the probabilistic interest-driven attention focusing (pIAF) model by extending the pLSA [42] suitable for our situation. The two steps are explained in the following subsections, respectively. 

It depends on content design whether AOF can be directly obtained by observing user gaze points on a catalog, and it is difficult in general. For example, when a user browses a catalog with food pictures, it cannot be obtained only from gaze points on which visual attributes the user focuses. The proposed method only requires ROIs decided based on each item (i.e., does not need to directly observe AOF) and therefore applicable to wide range of content design.

### AOF detection by short-term analysis

To detect the AOF (the red-highlighted area in Figure 2), we follow an anomaly detection approach: We first model neutral-browsing behavior and then utilize the likelihood of the model computed in each window position (temporal interval). Since a likelihood value tells us how likely an observation occurs under the model, the bias of the gaze behavior (i.e., distinctive gaze behavior) can be detected when the value falls below a predetermined threshold.

**Figure 3.**
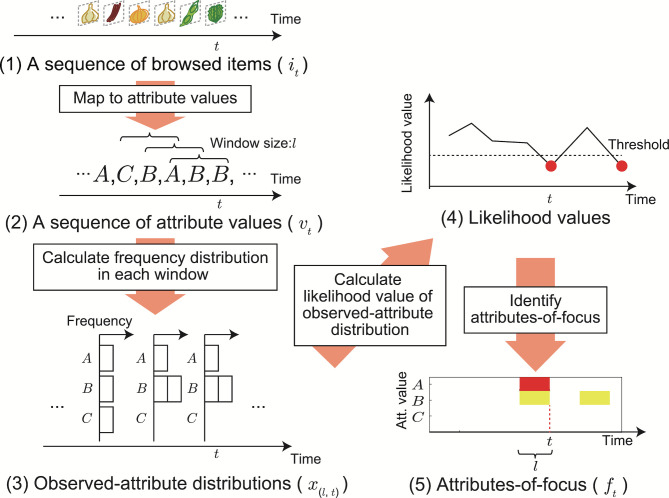
Flow of AOF detection using short-term analysis.

The flow of AOF detection is illustrated in Figure 3. Here we focus on analyzing the gaze-target transitions, i.e., the changes of the ROIs, although gaze duration also carries information. We first map a sequence of gaze points to a sequence of browsed items $\left(i_{1},\ldots ,i_{T}\right),i_{t}\in I$ based on item regions. Note that time t is defined based on the transitions between browsed items (i.e., $i_{t-1}\neq i_{t}$ ). For each attribute type a∈A , a sequence of attribute values of browsed items ($v_{1}^{\left(a\right)}$ , …$,v_{T}^{\left(a\right)}$ ) is obtained corresponding to the sequence of browsed items ((1) to (2) in Figure 3). Then, an analysis window with length l with shift size 1 is used for short-time analysis of the sequence. For each window position, a frequency distribution of attribute values, $x_{l}^{\left(a\right)}$ , is obtained for each attribute type a∈A ((2) to (3) in Figure 3); here, the sum of the total $K_{a}$ elements of $x_{l}^{\left(a\right)}$ is l . We refer to $x_{l}^{\left(a\right)}$ as an *observed-attribute distribution*. By assuming that each observed-attribute distribution $x_{l}^{\left(a\right)}$ in a neutral browsing obeys a probability distribution with function $g(x_{l}^{\left(a\right)};l,\phi^{\left(a\right)})$ , its likelihood value is calculated ((3) to (4) in Figure 3), where ϕ is the model parameter of neutral browsing described below. By comparing likelihood values with a threshold, periods of distinctive behavior are detected.

Neutral browsing is defined as browsing behavior in which the user does not focus on any specific attribute values. As a simple model for such behavior, we use a multinomial probability distribution described by

**(1):**
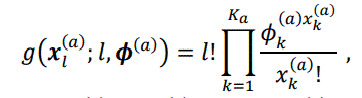

where $\phi^{\left(a\right)}=(\phi_{1}^{\left(a\right)},\ldots ,\phi_{K_{a}}^{\left(a\right)})$ . Here, $\phi_{k}^{\left(a\right)}$ denotes how likely the attribute value $V_{k}(a)$ is to be looked at under neutral browsing. Specifically, we determine the parameter as $\phi_{k}^{\left(a\right)}=N_{k}^{\left(a\right)}/N$ , where $N_{k}^{\left(a\right)}$ is the number of items that have $V_{k}^{\left(a\right)}$ , the k -th attribute value of attribute type a , in a catalog with N items. For the sake of simplicity, we ignore the effect of visual saliency (see also the related work section) in this model and assume that users browse catalog content uniformly when they are in neutral browsing, while its extension will be discussed later in the discussion section. 

Finally, the AOF are identified in each detected window interval by simply comparing the relative frequency of observed attribute values, $x_{l}^{\left(a\right)}/l$ , to the multinomial parameter $\phi^{\left(a\right)}$ . Specifically, if $x_{k}^{\left(a\right)}/l>\phi_{k}^{\left(a\right)}$ , attribute value $V_{k}^{\left(a\right)}$ is regarded as one of AOF ((4) to (5) in Figure 3). To simplify the AOF notation, we also use an indicator vector $f_{t}=\left(f_{t,1},\ldots ,f_{t,K}\right)\in {\left\{0,1\right\}}^{K}(K=\sum_{a\in A}K_{a})$ , of which element $f_{t,k}$ is 1 if the corresponding attribute value $V_{k}$ is detected as one of the AOF at time t , and 0 otherwise.

### Interest estimation using a generative model

**Figure 4.**
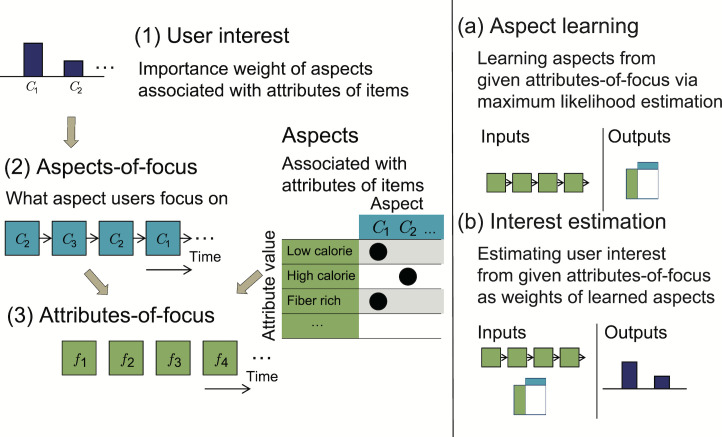
Illustration of interest-driven attention focusing model.

Modeling human behavior as a generative process using a probabilistic model is one approach to analyzing the internal states behind behavioral data. That is, the probabilistic model enables the model parameters to be estimated from data and to the infer internal states as latent parameters [22,23,24,25]. By borrowing this concept, we propose the pIAF model to learn the aspects and to estimate user interest (the green-highlighted area in Figure 2). Figure 4 illustrates the overview of the model.

The generative process to the AOF is modeled as follows (left side of Figure 4). Note that, while the green arrows in Figure 2 show the flow of aspect learning and interest estimation by the model, the direction of arrows in Figure 4 is the opposite because these arrows indicate the generative process of the AOF. During session s , a user is assumed to have interest θ(s) , which is considered to be the parameter of a categorical probability distribution $P\left(c_{t}=C_{r}|s\right)=\theta \left(s\right),r=1,\ldots ,R$ . We assume that the user first focuses on an aspect $c_{t}\in C_{r}$ (aspect-of-focus) (e.g., “healthy”) at each time t , where $c_{t}$ is determined in accordance with the distribution θ(s) ((1) to (2) in Figure 4). The user then turns his or her attention to some of the attribute values (e.g., “low calorie” and “fiber rich”) represented as the AOF $f_{t}$ , depending on the aspect-of-focus $c_{t}$ . When $c_{t}$ is determined to be $C_{r}$ , $f_{t}$ is assumed to obey the conditional probability distribution $P\left(f_{t}|c_{t}=C_{r}\right)=h(f_{t};n_{t},p_{r})$ ((2) to (3) in Figure 4). This is called an observation model, which will be discussed below. We assume that the user generates a sequence of AOF by repeating this generative process for every time step in the session s with the constant interest θ(s) .

Regarding observation model h , one can directly apply a categorical distribution similar to the original pLSA [42], and in fact, our previous work followed this option [11]. However, such a model seems to be unnatural as a user’s focus can take only a single attribute value at a time. In actual situations, a user jointly considers “multiple” attribute values in not all but “partial” attribute types. Therefore, the observation model h should be able to represent a joint distribution of attribute values constituting the AOF. 

To take both the multiplicity and partiality of users’ focus into account, we have extended the observation model to incorporate the concept of users’ attention resource [43]. Specifically, we consider a multinomial distribution on “all the attribute values” as the observation model and introduce the number of attribute values of simultaneous focus, $n_{t}$ , as a parameter of the attention resource at time t . Here, $h\left(f_{t};n_{t},p_{r}\right)$ is derived as

**(2):**
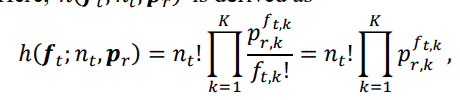

where $n_{t}=\sum_{k}f_{t,k}$ . Note that the parameter $n_{t}$ is assumed to be given as the number of detected AOF.

 When we have AOF sequences extracted from multiple sessions of decision making, the parameters of the proposed generative model, ${\left\{\theta \left(s\right)\right\}}_{s}$ (users’ interests) and ${\left\{p_{r}\right\}}_{r}$ (multinomial parameters that characterize aspects), can be estimated using maximum likelihood estimation similar to standard pLSA models (right side of Figure 4). The likelihood function in this problem is derived as follows from the probability that AOF $f_{t}$ in all the given sessions are observed (see Appendix for derivation):

**(3):**
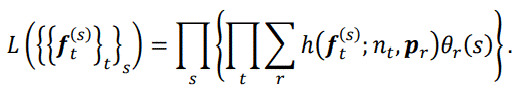

With this likelihood function, aspects can be learned as ${\left\{p_{r}\right\}}_{r}$ as shown in Figure 4 (a) through maximum likelihood estimation using observations in multiple sessions. The input and output parameters are summarized as follows (learning setting): 

Input: ${\left\{{\left\{f_{t}^{\left(s\right)}\right\}}_{t}\right\}}_{s}$ (a training set of AOF sequences); 

Output: ${\left\{p_{r}\right\}}_{r}$ (the degree of association between aspects and attribute values) and ${\left\{\theta \left(s\right)\right\}}_{s}$ (the users’ interests in training sessions). 

Meanwhile, the user’s interest $\theta \left(s\right)$ in the observed eye gaze data during session s can also be estimated once the aspect parameters are learned (Figure 4 (b)). Note that ${\left\{p_{r}\right\}}_{r}$ is given in this case, and therefore the input and output parameters of the estimation algorithm are summarized as follows (inference setting): 

Input: ${\left\{p_{r}\right\}}_{r}$ and ${\left\{f_{t}^{\left(s\right)}\right\}}_{t}$ (the AOF sequence during a session); 

Output: $\theta \left(s\right)$ (the user’s interest during the session).

## Evaluation

We evaluated the proposed two-step approach in terms of two perspectives. First, we investigated how the use of AOF detection affects the results of aspects obtained from eye gaze data compared with a method not using AOF detection. Then, we evaluated the accuracy of user interest estimation from gaze data for each decision-making session using the obtained aspects. 

To focus on evaluating the basic effectiveness of the proposed framework, we conducted an experiment in a controlled decision-making situation rather than in an actual situation, which involves a variety of decision-making factors. Specifically, during each session, we asked the participant to select one item from a set of items in a digital catalog in accordance with a particular requirement. We expected that the participants would compare several options with some bias regarding the attribute values of interest. Since each of the requirements can be characterized by several attribute values, the given tasks serve as the ground truth for quantitative evaluation of both the aspect learning and interest estimation.

### Participants

We conducted the experiment with the help of 37 participants (18 male and 19 female university students, ranging in age from 19 to 34, with a mean of 22.3 and a standard deviation of 2.9).

### Design

The importance of decision making depends on the content domain and affects the user’s behavior. For example, if the decision will greatly affect the user’s life (e.g., choosing a house), the user will examine the options more seriously and carefully than for less-important decisions (e.g., deciding what to eat for lunch). We therefore used the content domain of choosing a laptop computer, which is assumed to have moderate importance. 

**Figure 5.**
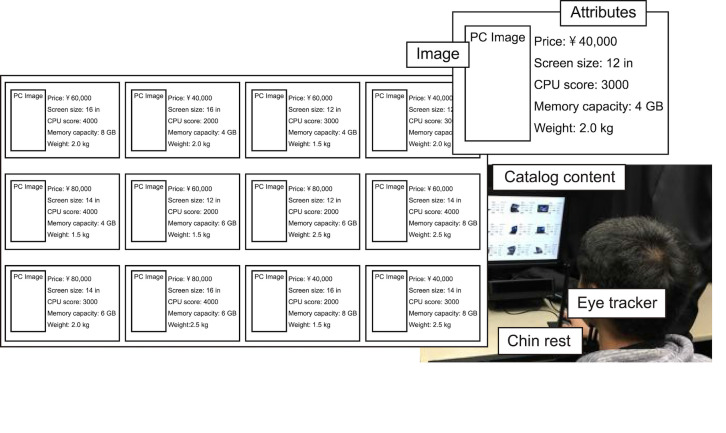
Experimental environment and displayed content of a catalog. Each item region consisted of a picture and descriptions of the PC attributes in text format. (Descriptions in this figure are translations from the original language, Japanese.)

As shown in Figure 5, the participants were asked to select a laptop computer (“PC”, hereinafter) from 12 PCs displayed on a screen. An eye tracker (Tobii X120) under the display was used to measure the participant’s eye movements, where the freedom of head movement was 300 x 220 x 300 mm, and the accuracy was 0.5 degrees. A sampling rate of 60 Hz (less than the maximum rate of 120 Hz) was used as we needed patterns of fixations rather than saccades. Each item region contained a written description of the attributes of the PC along with a picture to help the participants remember the position of the PC in the content. Each PC had five attribute types: *price, screen size, CPU score, memory capacity, *and* weight*. Each of the attribute types could take one of three values (e.g., low, middle, high). The content for each session was prepared so that 4 out of the 12 PCs had the same attribute value for each of the five types. To reduce the effect of the difference of content among the trials, we prepared three types of content by only replacing pictures of the PCs. That is, the sets of PCs’ attribute values in each content were the same. 

In each session, we gave a participant a task to select a PC that fulfills a specified requirement (situation and purpose). For simplicity, we hereinafter use “task” to also denote “requirement.” An example task is as follows: “Please assume that you will use your primary PC at home to watch movies and play games. Which PC do you think is the best for that situation?” We assumed that receiving a particular task would keep the participant’s interest $\theta \left(s\right)$ constant during the session. 

Three tasks common to all participants were prepared. Each participant completed three sessions corresponding to the three tasks, where the order of the tasks was systematically ordered in a Latin squares design. We expected that aspects obtained from eye gaze data would be related to the three tasks. In particular, we expected that the participant’s interest $\theta \left(s\right)$ took one of three states (1,0,0) , (0,1,0) , or (0,0,1) depending on the task, where $\theta_{r}=1$ for the aspect $C_{r}(r\in \left\{1,2,3\right\})$ that corresponds to the specified task. 

Each of these three tasks is implicitly related to several attribute values. For instance, a PC for playing games or watching movies requires “high CPU score and large memory capacity.” We refer to these values as *task-related attribute values*. Although the visual appearance conveys various types of information, the information actually obtained depended on the participant. Therefore, we designed that all the task-related attribute values were included in the text descriptions to equalize the amount of information given to the participants (Figure 5). Table 1 summarizes the three tasks and task-related attribute values. The aim of this experiment was to determine the learning capability of our framework to identify aspects common to multiple participants. Therefore, we designed tasks that could be easily and uniquely determined to some extent.

**Table 1:** Three tasks and task-related attribute values

Given tasks		Task-related attribute values
Situation	Purpose
Select a PC to use at home	To watch movies and play games	High CPU score, Large memory capacity
Select a PC to be carried around	To take notes	Small screen, Light
Select a PC at low cost	To view Web pages	Large memory capacity, Low price

We expected the participants to interpret each task as a set of attributes and then compare the options that satisfied the requirements. The participants’ knowledge affected not only the decision process [44] but also this interpretation. To set the participants’ knowledge almost equal, we briefly explained the meaning of the attribute types before explaining the tasks. Although this setting may seem too controlled, it is reasonable for our aim, which was not to determine the accuracy of detecting attribute values looked at by the participants but to determine the effect of AOF detection on aspect learning. 

To elicit the participants’ comparison behavior, we set the number of items that met the task to two so that the participants could not uniquely decide on one PC for the specified task. We randomized the positions of the PCs in the content to reduce the effect of the spatial layout. 

The total number of sessions in this experiment was 111 (37 participants x 3 tasks, contents).

### Procedure

Each participant was first asked to sit facing the display and to position his or her face in line with the chin rest (see Figure 5). After the calibrating the eye tracker, we explained the content and procedure of the experiment. The procedure in each session consisted of four steps:

Step 1. The participant was given a task (first two columns in Table 1). 

Step 2. The accuracy of the eye tracker’s calibrated parameters was confirmed. 

Step 3. The content was displayed, and the participant was asked to select one PC. 

Step 4. After the participant reported having made a selection, we asked which PC the participant had selected.

In Step 3, to explicitly separate the screening phase from the comparison phase [8,39], each item was displayed in turn at intervals of three seconds. Then they were all displayed and eye gaze was measured. Separating the screening phase in this way may have reduced the effect of the spatial position (see also the related work section), thereby enabling us to assume the participants browsed the content uniformly during neutral browsing. 

While items with a certain combination of attribute values (e.g., {low price, high CPU score, large memory capacity}) would satisfy more than one task, we did not include items with such combinations because once such an item appeared in a session, it could affect the comparison behaviors in the subsequent sessions due to the familiarity of the attribute combination. 

Because we did not limit the decision-making time, the participants browsed the content as long as they needed and had enough time to compare items before deciding on one.

### Results

**Figure 6.**
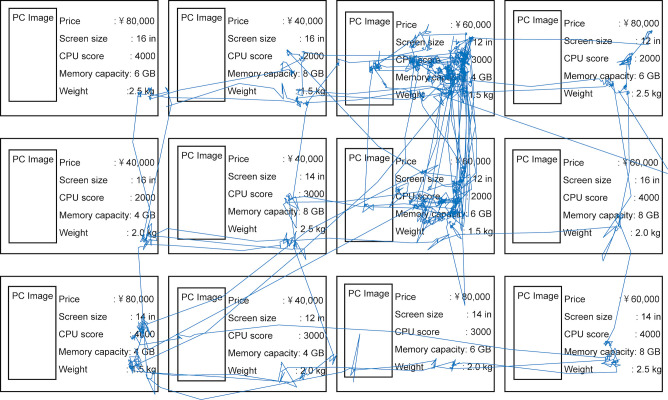
Example of gaze trajectory on the content. The descriptions in this figure are translations from the original Japanese same as Figure 5.

Figure 6 shows an example of gaze trajectory when a participant compared PCs by considering the attribute values of PCs. As can be seen in this figure, each participant mainly looked at and compared keywords of attribute values rather than pictures. Regarding the difference of the tasks, we confirmed from the obtained gaze data that the difference of both the physical duration and the number of items looked at in each task are small among the three tasks.

**Figure 7.**
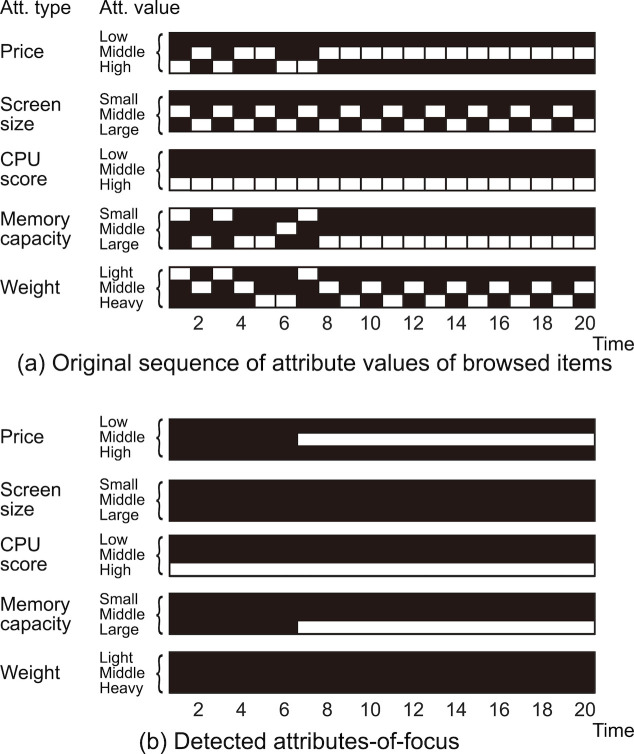
Example of detected AOF. (a) The original sequence of attribute values of browsed items; (b) detected AOF.

Example of AOF detected from actual eye gaze data are shown in Figure 7. Window size l and threshold value thr. used to determine the AOF were set to 5 and 0.03 , respectively. These parameters were determined experimentally to satisfy the following two constraints: At least one AOF was extracted from all sessions except for too short sessions; and, the only shared attribute values of the two items were extracted as AOF when a participant compared only two items in a short-term window. The task-related attribute values of this session were high CPU score and large memory capacity ( $V_{3}^{\left(CPU\right)}$ and $V_{3}^{\left(mem\right)}$ ; the notations were introduced in the methods section, and subscript 3 denotes the highest value for that attribute type). Remind that “Time” in the figure is based on the switching of gaze targets (i.e., ROIs). In this example, we can see that the participant first focused on high CPU scores ( $V_{3}^{\left(CPU\right)}$ ) and then compared items with not only a high CPU scores but also large memory capacity ( $V_{3}^{\left(CPU\right)}$ and $V_{3}^{\left(mem\right)}$ ) and a mid-range price ( $V_{2}^{\left(pri\right)}$ ).

**Figure 8.**
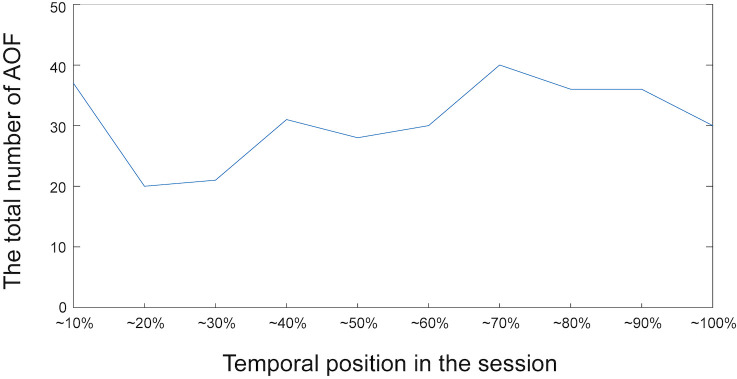
The total number of AOF detected in each temporal position in the session.

The compared items coincided with those we expected to be compared for the given task. An attribute value of midrange price ( $V_{2}^{\left(pri\right)}$ ) was also detected because the compared items commonly had the value $V_{2}^{\left(pri\right)}$ , although it was not included in the task. Similar choice behavior was seen in many other sessions with other participants. That is, the participants first focused on one task-related attribute value and then narrowed down the options by adding the other task-related attribute values to focus on. However, the participants did not always focus on attribute values related to their interests in the last couple of fixations. The total number of detected AOF in each (normalized) temporal position is depicted in Figure 8. In this plot, each session was divided into ten segments with respect to the temporal position, and the total number of AOF in each segment was calculated. This figure shows that, although the participants compared PCs mainly in latter part of the session, the comparison decreases in the end of the session. 

**Figure 9.**
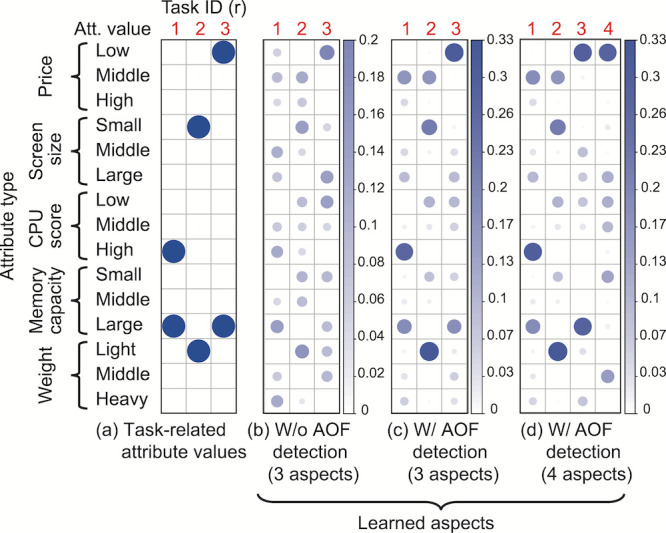
Task-related attribute values and learned aspects without and with AOF detection step using eye gaze data. In (b) and (c), the number of aspects was set to three (the same as that of the tasks) while it was set to four in (d). The size and color of each dot both depict the value of the multinomial parameter $p_{r,k}$, which represents the degree that aspect $C_{r}$ is associated with attribute value $V_{k}$ .

The results of the learned aspects without and with the AOF detection step are shown in Figure 9 (b) and (c), respectively. They can be compared qualitatively with the task-related attribute values shown in Figure 9 (a) in terms of the degree of association with the attribute values. The results without the AOF detection step (Figure 9 (b)) were obtained from original attribute-value sequences (e.g., Figure 7 (a)) while those with the AOF detection step (Figure 9 (c)) were obtained from sequences of detected AOF (e.g., Figure 7 (b)). In both cases, the number of aspects was set to three ( R=3 ), the same as that of the tasks, so that aspects corresponding to the given tasks were obtained.

These results show that the learned aspects were more distinct from one another when the AOF detection step was applied and that the attribute values highly associated with each aspect seem to be similar to the task-related attribute values for all three tasks. In addition, from the result with the different setting of the number of aspects, R=4 (Figure 9 (d)), we can see that task-related attribute values were still obtained successfully as aspects $C_{1}$ to $C_{3}$ despite the existence of another aspect, $C_{4}$ .

For quantitative evaluation of the effectiveness of the AOF detection step, the similarities between the learned aspects and the given tasks were calculated. The similarities were defined by the cosine similarity of two parameter vectors:

**(4):**
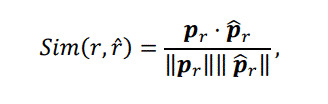

where $p_{r}(r=1,2,3)$ denotes the multinomial parameter of a learned aspect (see also the methods section) and ${\hat{p}}_{r}\in {\left\{0,1\right\}}^{K}(\hat{r}=1,2,3)$ is the vector form of the task-related attribute values in which an element is 1 if the value exists and 0 otherwise. Figure 10 shows the similarity scores between the task-related attribute values and the corresponding aspects found by matching the given tasks with the learned aspects. These results quantitatively show that the learned aspects were highly associated with the given task-related attribute values. 

**Figure 10.**
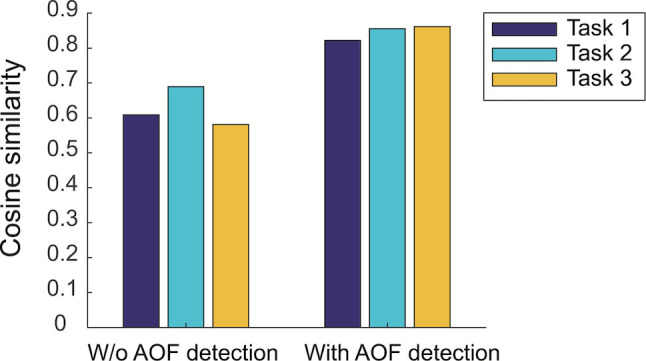
Similarities between task-related attribute values and learned aspects without and with AOF detection step.

To evaluate the accuracy of interest estimation, we conducted task estimation using the learned aspects, with the given task as the ground truth. Table 2 shows the results of task estimation based on the maximum probability of the estimated participants’ interest, ${argmax}_{r}\theta \left(s\right)=argmax_{r}P(C_{r}|s)$ . The interest $\theta \left(s\right)$ shown in Figure 9 (c) was estimated at the same time as aspect learning. That is, parameter estimation for the learning setting with Eq. (3) was used here since the main point of this evaluation is to confirm the effect of AOF detection on aspect learning and interest estimation. Note, on the other hand, that our approach can also be used to estimate user interest from newly observed gaze data with the inference setting. The accuracy of the task estimation was 83.8% . In 4 out of the 111 sessions, the AOF detection step did not detect the participant’s comparison behavior as biased gaze behavior due to quick decision making by the participants. That is, duration for those four sessions was less than 5, the size of the analysis window in the AOF detection step, l .

**Table 2:** Results of estimation of participants’ interest (engaging task). Number in each cell in column r(r=1,2,3) shows the number of sessions that were estimated as engaged task r . “No comparison” means the AOF detection step could not detect a bias of gaze behavior during the session.

		Estimated task			
		1	2	3	No comparison
	1	32	0	3	2
Actual task	2	0	30	7	0
	3	4	0	31	2

These results show the effectiveness of using the AOF detection step both in learning aspects of items and in estimating the participant’s engaging tasks (i.e., their interest) from their gaze behavior. In particular, the results shown in Figure 9 indicate that considering the participant’s “focus” on attributes (i.e., attributes-of-focus) is crucial to analyzing the participant’s comparison behavior.

## Discussion

The results presented above demonstrate the effectiveness of introducing the AOF detection step for learning aspects from gaze behavioral data and its effectiveness in estimating user interests reflected in gaze-target patterns. In this section, we discuss the limitations of our framework that arise from the assumptions made about the user internal model and observable gaze behavior.

### Dynamics of user interests

User interest is affected by many factors, including relatively stable individual preferences and temporary interests elicited by external information (e.g., novel information). However, our model assumes that a user’s interest is constant during a content browsing session. Although this assumption is useful for determining the basic capabilities of the proposed model and algorithm for learning aspects, which is the main focus of this paper, dynamic changes of user interest in actual situations should be addressed in order to put the proposed method into practical use. 

One approach to doing this is to introduce temporal segments by exploiting the results of the AOF detection step. By dividing AOF sequence into segments at the points where attribute values in the AOF change, we can consider that a user’s interest is constant in each segment. The proposed learning and estimation methods could then be applied to these segments, i.e., parameter vector θ could be assumed to be piecewise constant. While this segment-based model may cause shortage of training data, extending our pLSA-based model with a Bayesian approach such as latent Dirichlet allocation (LDA) [45] is a possible solution.

### Number of aspects

Since our method finds aspects on the basis of unsupervised learning as do topic models, the number of aspects needs to be given. In fact, the number of aspects was set to be the same as that of the tasks for the sake of evaluating the learned aspects in terms of the similarity to the given tasks. However, in practice, the number of aspects can vary depending on the user and the situation (e.g., the content and the task), so it is difficult to determine it beforehand in general. 

As shown by the results in Figure 9 (d), our approach is robust to the setting of the number to some extent. Moreover, the use of standard hyper-parameter estimation of unsupervised learning, e.g., non-parametric Bayes, should be effective in determining the appropriate number of aspects. However, the “interpretation” of learned aspects is desirable for many applications, such as speech dialog systems using aspects for probing questions [1], and needs to be investigated. 

### Temporal patterns of gaze targets

The temporal patterns of gaze targets convey useful information for estimating user interest during decision making. For example, if a user “re-fixates” on a target, he or she is probably specifically focusing on the target and comparing it with the alternatives [40]. Although these temporal patterns are indirectly considered in the AOF detection step to identify bias in the user’s gaze behavior in a short-term window, explicit modeling of gaze-transition patterns needs to be incorporated for a natural interpretation of user gaze behavior. 

### Information of gaze duration

Because we are particularly interested in user comparison behavior, changes in the gaze target (i.e., gaze-target transition) were used for the definition of time t in the proposed approach. This is suitable for treating the number of times a target is looked at as the weight placed on the target’s importance. However, this approach cannot be used to examine how carefully the user looks at each target. For example, Sugano et al. showed with random forests that duration is the feature that contributes the most to estimating the user’s interests[46]. Taking physical duration information into account may enable us to examine the degree of user focus on a gaze target and may increase the accuracy of the interest estimation. 

### Effect of visual saliency and content design

While we simply assumed that users uniformly browse displayed items on a screen during neutral browsing, visual saliency, such as salient regions and the position of items in a catalog, affects user gaze behavior (e.g., center bias [47]). Therefore, taking into account the effect of visual saliency in the neutral-browsing model may increase the accuracy of AOF detection. For example, a saliency map [36] can be used to determine the parameters of the multinomial distribution used in the neutral-browsing model as the weights on each attribute of items. 

In our experiment, the positions of the items were randomized to reduce the effect of item location in order to focus on the aspect-driven comparison behavior. However, the layouts in actual catalog content are not randomized but rather structured (e.g., similar items are arranged more closely together). This affects content browsing and is actually useful for analyzing browsing states in a decision-making process [40]. Therefore, the information of content design needs to be considered for interest estimation.

## Conclusion

This paper addressed the problem of finding a representation for a user interest and its estimation from eye gaze data in digital-catalog browsing using a data-driven approach. By introducing *aspects* as approximate representations of user values when choosing items, we aimed at obtaining a set of aspects from eye gaze data using unsupervised learning of the pIAF model, a probabilistic generative model of attributes-of-focus (AOF). The main contribution of this paper is the introduction of an AOF detection step to overcome the problem of such data-driven learning and estimation being strongly affected by item information and attributes of no interest to the user. We evaluated the validity of this approach with actual eye gaze data and found that it successfully constructs distinctive aspects highly correlated with user decision goals compared with an approach without the AOF detection step. Future work includes investigating ways to overcome the limitations discussed in the previous section and applying the proposed method to interactive systems that can proactively assist user decision making.

### Ethics and Conflict of Interest

The authors declare that the contents of the article are in agreement with the ethics described in http://biblio.unibe.ch/portale/elibrary/BOP/jemr/ethics.html and that there is no conflict of interest regarding the publication of this paper. 

### Acknowledgements

This work was supported by JSPS KAKENHI Grant Numbers JP15J06965, JP26280075, JP19H04226, and JST, PRESTO JPMJPR14D1.

## Appendix

### Derivation of the equation for maximum likelihood estimation

The joint probability of an aspect-of-focus and an attribute-of-focus during session s is derived as follows by assuming the conditional independence of $f_{t}$ and s given $c_{t}$ :

Hence, the probability that an attribute-of-focus $f_{t}$ is observed during session s is given by

When we have S sessions of decision making, the parameters to be estimated are ${\left\{\theta \left(s\right)\right\}}_{s}$ , users’ interests, and ${\left\{p_{r}\right\}}_{r}$ , which are multinomial parameters that characterize aspects. Here, the probability of an attribute-of-focus $f_{t}$ during session s is given by Eq. (6), where parameter $n_{t}$ is given as described in the methods section. Given parameters $\theta \left(s\right)$ and ${\left\{p_{r}\right\}}_{r}$ , the likelihood of a sequence of attributes-of-focus ${\left\{f_{t}^{\left(s\right)}\right\}}_{t}$ during session s is computed as

By computing the likelihood of all sessions, we can obtain Eq. (3).

The model parameters can therefore be estimated by solving the following optimization problem derived by maximizing the logarithm of Eq. (3):

This problem can be solved using an expectation-maximization algorithm similar to parameter estimation of pLSA.
